# Practice variation in timing of antenatal corticosteroid administration in early‐onset fetal growth restriction: A secondary analysis of the Dutch STRIDER study

**DOI:** 10.1111/aogs.14692

**Published:** 2023-10-30

**Authors:** Leah I. Prins, Mette van de Meent, Judith Kooiman, Anouk Pels, Sanne J. Gordijn, Titia Lely, Wessel Ganzevoort

**Affiliations:** ^1^ Department of Obstetrics and Gynecology Amsterdam University Medical Centers, University of Amsterdam Amsterdam The Netherlands; ^2^ Amsterdam Reproduction and Development Research Institute Amsterdam The Netherlands; ^3^ Department of Obstetrics and Gynecology University Medical Center Utrecht Utrecht The Netherlands; ^4^ Department of Obstetrics and Gynecology University Medical Center Groningen Groningen The Netherlands

**Keywords:** high risk pregnancy, preeclampsia, prenatal care, prenatal diagnosis, preterm birth

## Abstract

**Introduction:**

In early‐onset fetal growth restriction the fetus fails to thrive in utero due to unmet fetal metabolic demands. This condition is linked to perinatal mortality and severe neonatal morbidity. Maternal administration of corticosteroids in high‐risk pregnancies for preterm birth at a gestational age between 24 and 34 weeks has been shown to reduce perinatal mortality and morbidity. Practice variation exists in the timing of the administration of corticosteroids based on umbilical artery monitoring findings in early‐onset fetal growth restriction. The aim of this study was to examine differences in neonatal outcomes when comparing different corticosteroid timing strategies.

**Material and methods:**

This was a post‐hoc analysis of the Dutch STRIDER trial. We examined neonatal outcomes when comparing institutional strategies of early (umbilical artery pulsatility index >95th centile) and late (umbilical artery shows absent or reversed end‐diastolic flow) administration of corticosteroids. The primary outcomes were neonatal mortality and a composite of neonatal mortality and neonatal morbidity, defined as bronchopulmonary dysplasia, intraventricular hemorrhage, necrotizing enterocolitis or retinopathy of prematurity. We also analyzed predictors for adverse neonatal outcomes, including gestational age at delivery, birthweight, maternal hypertensive disorders, and time interval between corticosteroids and birth.

**Results:**

A total of 120 patients matched our inclusion criteria. In 69 (57.5%) the early strategy was applied and in 51 (42.5%) patients the late strategy. Median gestational age at delivery was 28 4/7 (± 3, 3/7) weeks. Median birthweight was 708 (± 304) g. Composite primary outcome was found in 57 (47.5%) neonates. No significant differences were observed in the primary outcome between the two strategies (neonatal mortality adjusted odds ratio [OR] 1.22, 95% CI 0.44–3.38; composite primary outcome adjusted OR 1.05, 95% CI 0.42–2.64). Only gestational age at delivery was a significant predictor for improved neonatal outcome (adjusted OR 0.91, 95% CI 0.86–0.96).

**Conclusions:**

No significant differences in neonatal outcomes were observed when comparing early and late strategy of antenatal corticosteroid administration on neonatal outcomes in pregnancies complicated by early‐onset fetal growth restriction. We found no apparent risk contribution of interval between corticosteroid administration and delivery in multivariate analysis. Gestational age at delivery was found to be an important predictor of neonatal outcome.

## Graphical abstract

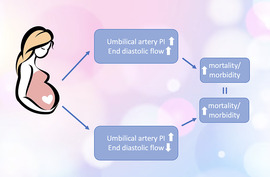

**Abbreviations**
A/REDF: absent/reversed end diastolic flow
BPD: bronchopulmonary dysplasia
CCS: corticosteroids
CI: confidence interval
eoFGR: early onset fetal growth restriction
FGR: fetal growth restriction
IQR: interquartile range
IVH: intraventricular hemorrhage
NEC: necrotizing enterocolitis
OR: odds ratio
PI: pulsatility index
PVL: periventricular or cystic leukomalacia
RDS: respiratory distress syndrome
REDF: reversed end diastolic flow
ROP: retinopathy of prematurity
SD: standard deviation
UA: umbilical artery

**Key message**

“Early” vs “late” antenatal corticosteroids gave no different neonatal outcomes. Short (<7 days) or long (>7 days) interval of corticosteroids before birth gave no different outcomes. Gestational age at delivery was a significant predictor for improved neonatal outcomes.

## 1 INTRODUCTION

Early‐onset fetal growth restriction (eoFGR) is associated with severe perinatal morbidity and mortality, and largely coincides with the maternal syndrome of early‐onset preeclampsia.^1^, ^2^ Early onset FGR (onset <32 weeks of gestation) is defined as an abdominal circumference <3rd centile, an estimated fetal weight <3rd centile or absent or reversed end‐diastolic flow (A/REDF) in the umbilical artery (UA), or a combination of contributory parameters defined as an estimated fetal weight or abdominal circumference <10th centile combined with a pulsatility index (PI) of the umbilical or uterine artery >95th centile.^3^

Insufficient feto‐maternal exchange in the placenta is the underlying pathophysiologic mechanism of eoFGR. The typical placental lesion is maternal vascular malperfusion. When the fetal metabolic and gaseous demands are insufficiently met, the fetus fails to develop and thrive in utero. An important step in the prevention of stillbirth is expedited delivery when fetal hypoxia develops.^4^

Antenatal administration of corticosteroids in pregnant women at risk for (spontaneous) premature birth between 24 and 34 weeks has been shown to reduce neonatal mortality and morbidity, particularly respiratory distress syndrome (RDS), bronchopulmonary dysplasia (BPD), intraventricular hemorrhage (IVH), periventricular or cystic leukomalacia (PVL) and necrotizing enterocolitis (NEC).^5^, ^6^, ^7^ Post‐hoc analyses of studies in spontaneous preterm birth suggest that administration of corticosteroids within 7 days before birth results in the largest treatment effect, ie a higher decrease in mortality and morbidity when compared with administration at an interval of more than 7 days before birth.^7^, ^8^, ^9^ This suggests that it is important to adequately time a single course of antenatal corticosteroid injections.^10^

Delivery based on imminent fetal hypoxia is unpredictable and for that reason clinicians use precursor monitoring variables that inherently have a lead‐time.^11^ In the Netherlands, practice variation exists in the triggers for administration of corticosteroids.^12^ The early strategy comprises administration of corticosteroids when the UA PI exceeds the 95th centile and the late strategy when an A/REDF in the UA is observed. Comparative evidence of both approaches is lacking. Therefore, international guidelines do not provide guidance on which (ultrasound) parameters should prompt corticosteroid administration in eoFGR.^13^ In this study, we used Dutch practice variation to explore the impact of timing of antenatal corticosteroid administration on neonatal outcomes in a post‐hoc analysis of the Dutch STRIDER cohort: a prospective randomized controlled trial in women with severe eoFGR.

## 2 MATERIAL AND METHODS

This was a post‐hoc analysis of the Dutch STRIDER (Sildenafil TheRapy In Dismal prognosis Early onset fetal growth Restriction) trial.^14^ Methods and results of the trial are extensively described in the main publication. In short, the Dutch STRIDER trial was a multicenter placebo‐controlled randomized controlled trial investigating the effect of Sildenafil on perinatal mortality and morbidity, compared with placebo. Pregnant women with eoFGR were eligible for inclusion between 20+0 weeks and 29+6 weeks of gestation. Early onset FGR was defined as fetal abdominal circumference <3rd percentile or the estimated fetal weight <5th percentile, combined with either unilateral or bilateral notching of the uterine artery, PI of the UA >95th percentile or PI of the middle cerebral artery <5th percentile. Study participants were randomized to receive either sildenafil 25 mg three times daily or placebo three times daily. The primary outcome of the Dutch STRIDER trial was a composite of death or major neonatal morbidity assessed at hospital discharge, defined as IVH grade three or more, PVL grade two or more, moderate, severe BPD or NEC grade two or more, retinopathy of prematurity (ROP) treated by surgery, or laser therapy. The trial was stopped early because of significantly higher occurrence of persistent pulmonary hypertension of the neonate in the sildenafil group in the face of futility.

Timing of maternal corticosteroid treatment was according to hospital protocol and not prescribed in the STRIDER trial protocol. For this secondary analysis, we used practice variation in timing of corticosteroid administration between the participating hospitals to compare neonatal outcomes between the “early” and “late” strategy. Hospitals were designated as “early” when local protocol was to administer corticosteroids when the pulsatility index of the UA exceeded the 95th percentile. If local protocols were prescribed to initiate antenatal corticosteroid treatment when the UA showed absent or reversed end‐diastolic flow, or signs of fetal compromise, they were designated as “late” strategy.

Secondly, we explored independent predictors for adverse neonatal outcomes, including gestational age at delivery, birthweight, maternal hypertensive disorders and whether or not the administration of corticosteroids was within 7 days before birth. In patients who received a second course of corticosteroids, a so‐called “corticosteroid rescue course”, the time between the last corticosteroid course and birth was used for analysis. A complete course of corticosteroids was defined as two intramuscular injections with 24 hours in between and at least 48 hours after the first injection.

### 2.1 Participants

From the original cohort, we selected women who had a live birth after 24+0 weeks of gestation who received corticosteroids during their pregnancy for anticipated prematurity.

Neonates with congenital infections or malformations at birth (unknown at the time of inclusion in the study) were excluded from our analysis, as this could possibly interfere with the neonatal outcomes.

### 2.2 Outcomes

The primary outcomes for the current analysis were neonatal mortality and a composite of neonatal mortality and survival with major neonatal morbidity, defined as any of the following morbidities: moderate or severe BPD, IVH grade three or more, PVL grade two or more, the presence of NEC Bell's stage two or more and ROP for which laser therapy was indicated (similar to the original STRIDER trial analysis). We also examined whether adding RDS to the primary outcome composite changed results. Next, a composite of RDS and BPD was examined. Individual neonatal morbidities were analyzed separately as well as RDS in secondary outcomes.

### 2.3 Statistical analyses

Baseline characteristics were reported as mean with standard deviations (SD), median with interquartile ranges (IQR) or numbers with percentages.

The first analysis was on an “intention‐to‐treat” basis and compared the primary and secondary outcomes between patients treated in hospitals using the “early” vs. “late” corticosteroid strategy. Primary outcomes were compared between the “early” and “late” groups by performing univariate logistic regression analysis, with results reported as odds ratios (ORs) with corresponding 95% confidence intervals (CI). At a second stage, these ORs were corrected for gestational age at birth and birthweight as possible confounders using multivariate logistic regression. A sensitivity analysis was performed repeating the above analysis by excluding women with a maternal indication for delivery, as this might influence both adherence to corticosteroid administration protocols and the primary outcome.

The second sensitivity analysis was on a “per‐protocol” basis and compared the primary outcome between patients who received corticosteroids at the early stage vs those who received corticosteroids at the late stage. Again, statistical analyses as stated above were performed.

Predictors of the primary outcome were analyzed using univariate and multivariate logistic regression and reported as OR with 95% CI. The ORs were then corrected for interval between corticosteroid administration in days on a continuous scale, as well as an interval of equal or less or an interval of >7 days between last corticosteroid course administration and birth.

Statistical significance was set at a two‐sided *p*‐value of <0.05. All statistical analyses were conducted using statistical software IBM SPSS Statistics Data Editor version 26.0.0.1.^15^

### 2.4 Ethics statement

This study is a secondary analysis of a randomized controlled trial where informed consent was obtained.^14^ Methods are extensively described in the main publication.

## 3 RESULTS

**FIGURE 1:**
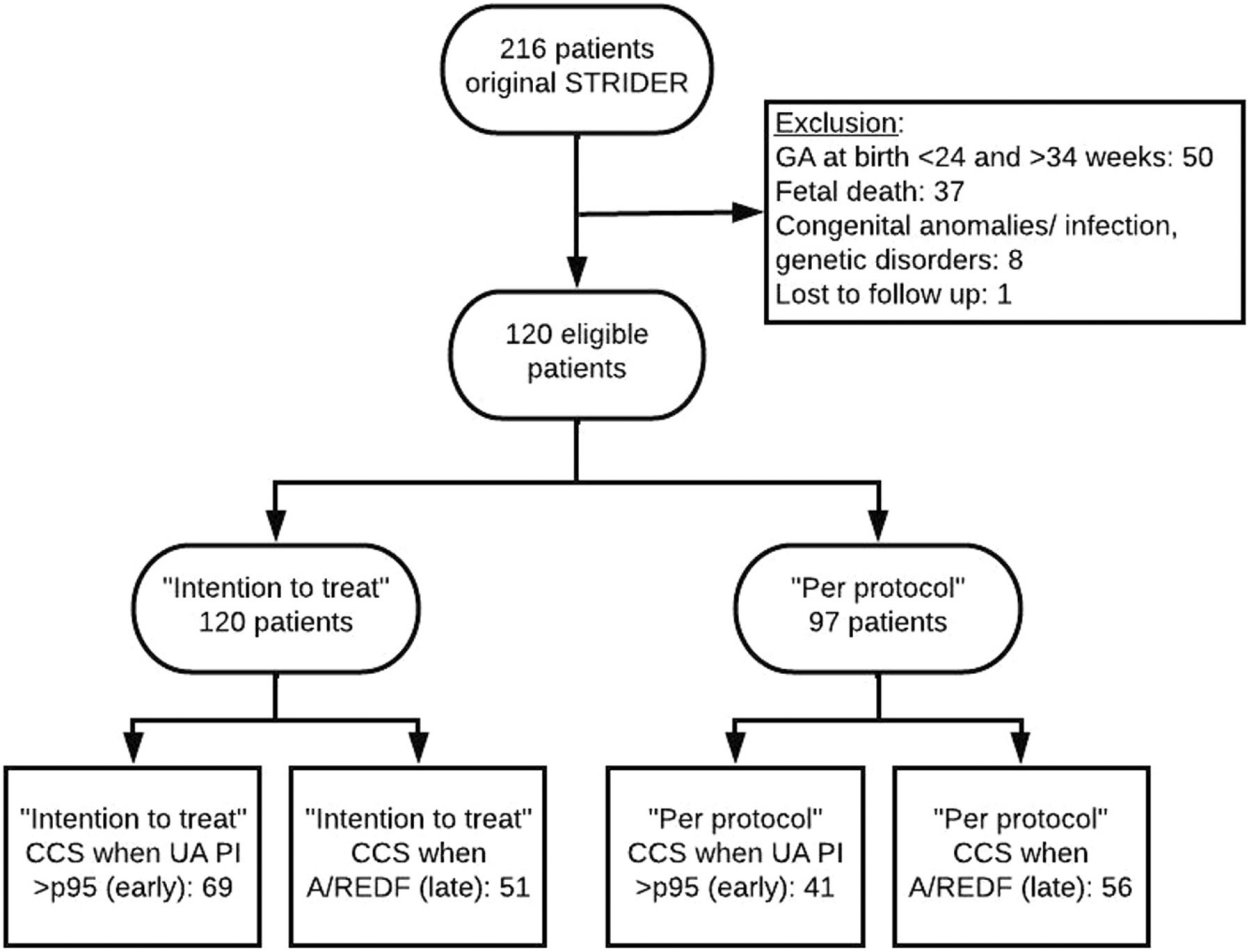
Flowchart of study. A/REDF, absent or reversed end diastolic flow; CCS, corticosteroids; GA, gestational age; PI, pulsatility index; UA, umbilical artery.

In the Dutch STRIDER trial, 216 pregnant women were randomized at 11 different sites (Figure S1). One hundred twenty participants fulfilled the inclusion criteria for the current analysis (Figure 1). Reasons for exclusion in the current analysis are displayed in Figure S1.

Baseline characteristics are presented in Table 1, showing data of all 120 patients, and separately for patients treated with corticosteroids (CCS) based on “intention to treat” basis for an early or a late strategy.

**TABLE 1:** Study characteristics of total population and separately per treatment group with intention‐to‐treat approach, *n* = 120.

	*n*/total *n*, %		
	Total group (*n* = 120)	ITT early strategy^a^ (*n* = 69)	ITT late strategy^b^ (*n* = 51)
Age, years, mean (SD)	31.4 (4.7)	30.5 (4.8)	32.7 (4.2)
BMI, mean (SD)	25.8 (5.4)	25.7 (5.4)	25.9 (5.6)
Ethnicity (missing = 1)			
Caucasian (white)	99 (83.2)	54 (78.3)	45 (90.0)
African (non‐white)	6 (5.1)	2 (2.9)	4 (8.0)
Asian	4 (3.4)	4 (5.8)	0 (0.0)
Other	10 (8.4)	9 (13.0)	1 (2.0)
Maternal smoking (missing = 2)			
Non‐smoker	95 (80.5)	53 (79.1)	42 (82.4)
Stopped before GA 15+0 weeks	6 (5.1)	4 (6.0)	2 (3.9)
Stopped after GA 15+0 weeks	4 (2.5)	2 (3.0)	1 (2.0)
Current smoker	14 (11.9)	8 (11.9)	6 (11.8)
Multipara	43 (35.8)	24 (34.8)	19 (37.3)
Data at inclusion			
GA, mean (SD)	25 1/7 (2 0/7)	24 2/7 (2 1/7)	25 0/7 (1 5/7)
Pulsatility index			
Umbilical artery >95th percentile (missing = 6)	90 (78.9)	46 (73.0)	44 (86.3)
MCA <5th percentile (missing = 3)	60 (51.3)	33 (50.0)	27 (52.9)
End‐diastolic flow (missing = 7)			
Positive	66 (58.4)	39 (61.9)	27 (54.0)
Absent	36 (31.9)	18 (28.6)	18 (36.0)
Reversed	11 (9.7)	6 (9.5)	5 (10.0)
Gestational hypertension	28 (23.3)	17 (24.6)	11 (21.6)
Preeclampsia or HELLP syndrome	40 (33.3)	24 (34.8)	16 (31.4)
Use of antihypertensive drugs	52 (43.3)	25 (36.2)	27 (52.9)
Allocated to sildenafil	64 (53.3)	38 (55.1)	26 (51.0)
Male fetus	60 (50.0)	34 (49.3)	26 (51.0)
GA at last CCS administration, weeks [IQR]	27 0/7 [2 5/7]	27 0/7 [2 5/7]	27 5/7 [2 6/7]
Interval CCS and birth <7 days	63 (52.5)	34 (49.3)	29 (56.9)
Repeated CCS course (“rescue course”)	12 (10.0)	9 (13.0)	3 (5.9)
Mode of delivery, cesarean section	113 (94.2)	65 (92.4)	48 (94.1)
Gestational age at delivery, weeks [IQR]	28 4/7 [3 3/7]	28 3/7 [4 0/7]	28 5/7 [3 3/7]
Birthweight, g (IQR)	707.5 [304]	710.0 [353]	700.0 [295]
Maternal indication for delivery (missing = 4)	24 (12.0)	14 (20.6)	10 (20.8)
Protocol adherence^c^ (missing = 23)	50 (51.5)	25 (44.6)	25 (61.0)

Abbreviations: BMI, body mass index; CCS, corticosteroids; FGR, fetal growth restriction; GA, gestational age; HELLP, hemolysis elevated liver enzymes low platelets; IQR, interquartile range; ITT, intention‐to‐treat; MCA, middle cerebral artery.

^a^ Early strategy: corticosteroids when UA PI >95 percentile or MCA PI <5 percentile.

^b^ Late strategy: corticosteroids when absent or reversed end diastolic flow of UA.

^c^ Percentage of patients treated according to prespecified protocol for early or late strategy.

The median gestational age at birth was 28 4/7 (±3.3/7) weeks. The median birthweight was 708 (±304) grams (Table 1). Induction of labor was initiated in 24 (12.0%) women on maternal indication (and not fetal indication), mainly because of worsening maternal clinical condition.

Fifty‐seven (47.5%) neonates fulfilled the criteria of the primary outcomes (Table 2). No neonates were diagnosed with PVL stage two or more. In 20 patients, a completion of the corticosteroid course was not achieved. In the patient group with the late strategy, seven (13.7%) patients did not complete the course vs 13 (18.8%) in the early group. Of all patients, 12 (10.0%) received a “rescue course” of corticosteroids (Table 1).

**TABLE 2:** Study outcomes comparing early vs late strategy on intention‐to‐treat basis.

Outcome	Early strategy^a^ *n*, % (*n* = 69)	Late strategy^b^ *n*, % (*n* = 51)	Crude OR (95% CI)	Adjusted OR (95% CI)^c^
Primary outcomes				
Neonatal mortality (*n* = 23)	13 (18.8)	10 (19.6)	1.05 (0.42–2.63)	1.22 (0.44–3.38)
Composite primary outcome (*n* = 57)	33 (47.8)	24 (47.1)	0.97 (0.47–2.00)	1.05 (0.42–2.64)
Secondary outcomes				
NEC stage 2 or more (*n* = 14)	7 (10.1)	7 (13.7)	1.21 (0.41–3.59)	1.47 (0.18–10.18)
IVH grade 3 or more (*n* = 4)	2 (3.0)	2 (4.2)	1.39 (0.19–10.24)	1.35 (0.12–11.34)
Moderate or severe BPD (*n* = 34)	19 (32.2)	15 (34.1)	1.09 (0.48–2.50)	1.20 (0.43–3.37)
ROP requiring laser therapy (*n* = 9)	7 (30.4)	2 (28.6)	0.91 (0.14–5.90)	1.19 (0.12–11.34)
RDS (*n* = 71)	40 (58.0)	31 (60.8)	1.12 (0.54–2.35)	1.18 (0.42–3.32)

*Note*: Primary outcome: composite of neonatal mortality, moderate or severe BPD, IVH grade >2, NEC Bell stage >1 and ROP with laser therapy.

Abbreviations: BPD, bronchopulmonary dysplasia; IVH, intraventricular hemorrhage; NEC, necrotizing enterocolitis; OR, odds ratio; RDS, respiratory distress syndrome; ROP, retinopathy of prematurity.

^a^ Early strategy: corticosteroids when UA PI >95 percentile or MCA PI <5 percentile.

^b^ Late strategy: corticosteroids when absent/reversed end diastolic flow of UA.

^c^ Adjusted for gestational age at delivery and birthweight.

### 3.1 Comparison early vs late strategy—intention‐to‐treat analysis

Of the 11 hospitals participating in the STRIDER trial, seven hospitals (*n* = 69 patients) followed the early and three hospitals (*n* = 51 patients) the late strategy on corticosteroid administration. One hospital only included patients that did not meet the inclusion criteria of this analysis (Table 2).

In the intention‐to‐treat analysis, no significant differences were observed in the primary outcomes (neonatal mortality adjusted OR [aOR] 1.22, 95% CI 0.44–3.38; neonatal mortality or major neonatal morbidity aOR, 1.05, 95% CI 0.42–2.64) between women treated in hospitals adhering to the early or the late strategy. Secondary outcomes of the individual morbidities NEC, IVH, BPD and ROP also did not differ significantly between the two strategies (Table 2). When adding RDS to the primary outcome composite, aORs lowered but were still not significant (aOR 0.84, 95% CI 0.27–2.62). Furthermore, when analyzing RDS combined with BPD as outcome, aORs were also not significant (aOR 1.76, 95% CI 0.58–5.35). Data are not presented.

Sensitivity analysis excluding patients with induction of labor or cesarean section on maternal indication, also showed no significant differences (Table S2).

### 3.2 Comparison early vs late strategy – per protocol analysis

For the sensitivity analysis we explored the timing of CCS in relationship with the actual monitoring findings. A total of 97 patients received CCS when UA PI >95th percentile or when A/REDF of the UA was observed. Reasons for missing data are explored in Table S1. In 41 (42.2%) patients, CCS were administered when UA PI >95th percentile (but with PEDF) was measured. In 56 (57.8%) patients, CCS were given when A/REDF of the UA was seen. No significant differences were observed in the primary outcomes (neonatal mortality aOR 0.97, 95% CI 0.33–2.86; neonatal mortality or major neonatal morbidity aOR 0.78, 95% CI 0.28–2.19) between women receiving CCS when the UA PI exceeded the 95th percentile and women receiving CCS when A/REDF was measured. Secondary outcomes also did not significantly differ between these patient groups (Table S3).

### 3.3 Analysis of predictors

Only gestational age at delivery was a significant predictor (aOR 0.91, 95% CI 0.86–0.96) for the primary outcome, the lower the worse (Table 3).

**TABLE 3:** Predictors of composite primary outcome.

Predictor	Crude OR (95% CI)	Adjusted OR (95% CI)^b^	Adjusted OR (95% CI)^c^
Gestational age at delivery, days	0.90 (0.87–0.94) *P* = <0.01	0.91 (0.86–0.96) *P* = <0.01	0.92 (0.87–0.97) *P* = <0.01
Birthweight, g	0.99 (0.99–1.00) *P* ≤0.01	1.00 (0.99–1.00) *P* = 0.07	1.00 (0.99–1.00) *P* = 0.08
Maternal hypertensive disease^a^	1.58 (0.77–3.26) *P* = 0.21	0.62 (0.23–1.75) *P* = 0.37	0.68 (0.24–1.91) *P* = 0.46
Interval between CCS and birth (continue)^b^	0.95 (0.91–0.98) *P* = 0.01	1.04 (0.98–1.10) *P* = 0.22	NA
Interval between CCS and birth (grouped)^c^	0.38 (0.18–0.80) *P* = 0.01	NA	1.33 (0.48–3.69) *P* = 0.58

Abbreviations: CCS, corticosteroids; NA, not applicable; OR, odds ratio.

^a^ Maternal disease composite of hypertensive disorder, preeclampsia and HELLP.

^b^ Interval between CCS and birth on a continuous scale, adjusted for all other predictors.

^c^ Interval between CCS and birth assessed using a cut‐off of 7 days, adjusted for all other predictors.

Birthweight, maternal hypertensive disorders, interval from corticosteroids until birth on a continuous scale or dichotomized (interval ≤ or interval >7 days) were not predictive of the primary outcome.

## 4 DISCUSSION

In this post‐hoc analysis of a randomized controlled trial, we found no differences in neonatal outcomes of strategies for eoFGR fetuses in whom corticosteroids were administered once UA Doppler measurements were >95th percentile (early strategy) compared with administration when A/REDF of the UA was observed (late strategy). In an intention‐to‐treat approach, the interval between antenatal corticosteroid administration and delivery did not contribute to prediction in multivariable analysis. There is scarce literature to compare our findings with, since head‐to‐head comparisons of strategies are lacking. In previous post‐hoc analyses of studies that excluded growth‐restricted neonates, it has been reported that the preferred timing of antenatal corticosteroids is between 1 and 7 days before birth.^16^

There are no specifications in current guidelines regarding the timing of the administration of corticosteroids other than “women between 24+0 and 33+6 weeks of gestation in whom imminent preterm birth is anticipated”, as quoted from RCOG guideline concerning antenatal corticosteroids.^17^ The advice given in other guidelines is similar.^18^, ^19^, ^20^ The lack of more specific timing advice may be because the route to iatrogenic birth is so different in this syndrome with unpredictable progression of fetal and maternal risks.^11^, ^21^ Our data show no differences in primary outcomes whether corticosteroids are administered close to or long time before birth. However, only around 50% of our patient group delivered within 7 days after CCS administration, which makes the sample sizes rather small. This could result in underestimation of our final results on primary outcomes. Nevertheless, it could then be argued that waiting until A/REDF of the UA and, as a consequence, minimizing the time between CCS and birth, might not be beneficial because it increases the chances of an incomplete dosage.

Another approach to the interpretation of these results could be that CCS do not have the same beneficial effect in this particular patient group as they had in the studies on spontaneous preterm birth.^22^ The theory has been postulated that increased endogenous corticosteroid production associated with chronic intrauterine stress in FGR fetuses already enhances lung maturation.^21^, ^23^, ^24^ No randomized studies examining this effect in FGR are available and subgroup analyses on the FGR population have not been performed.^25^ Nevertheless, in animal studies, positive effects of corticosteroids in FGR animals on lung maturation were described.^26^, ^27^ To determine whether there is an effect in human FGR fetuses, we plan to perform an individual patient data meta‐analysis to inform a definitive randomized trial. Alternatively, a contributing factor to an absence of effect may be that improved neonatal management has reduced the absolute effects of antenatal corticosteroids altogether, rendering timing of less importance.

The absence of an effect in this study could be linked to several sources of bias. First, residual confounding from institutional differences including population mix or other institutional policies may obscure a true effect. However, we find no such indication in the baseline characteristics in both groups.

Secondly, lack of power may contribute to the null hypothesis, partly due to missing data. However, the numbers of patients were comparable with earlier studies.^27^, ^28^ Also, clinically relevant differences (irrespective of their statistical significance) were absent between the early and the late strategy. From the current results the true effect is unlikely to be as large as in the trials of corticosteroid therapy, which mainly included anticipated spontaneous preterm birth.

Thirdly, there was reduction in the number of women with absent end diastolic flow in the early strategy group and vice versa; however, no indication of an important influence in the “per‐protocol” analysis was observed.

## 5 CONCLUSION

Based on this post‐hoc analysis of data of the Dutch STRIDER trial, and on the existing literature, it is yet inconclusive whether antenatal corticosteroids in eoFGR fetuses improve outcomes and, if so, what the ideal timing of administration is. A randomized controlled trial however, may only be feasible and ethical if a meta‐analysis with individual patient data of all available trials on this topic remain inconclusive. Therefore, we first plan to perform an individual patient data meta‐analysis.

## AUTHOR CONTRIBUTIONS

WG, SJG and TL: conceptualization. AP, LIP: data curation, methodology and formal analysis. LIP: methodology, writing – original draft and visualization. WG, SJG, TL, JK, MvdM and LIP: supervision and writing – review and editing.

## FUNDING INFORMATION

Netherlands Organization for Health Research and Development (project No. 836021023).

## CONFLICT OF INTEREST STATEMENT

The authors have stated explicitly that there are no conflicts of interest in connection with this article.

## Supporting information


Figure S1.
Table S1–S3.
