# Supplementary material for: Practice variation in timing of antenatal corticosteroid administration in early‐onset fetal growth restriction: A secondary analysis of the Dutch STRIDER study
Source: Acta Obstet Gynecol Scand. 2023 Oct 30;103(1):77–84. doi: 10.1111/aogs.14692 (PMC10755118; doi:10.1111/aogs.14692)
Supplement: Supplementary file 1 — Figure S1. Table S1–S3. [file AOGS-103-77-s001.docx]

**Supporting Information**

Table S1. Reasons for missing data

|  | |
| --- | --- |
| Reasons missing protocol adherence (N=23) | Number, percentage % |
| Maternal indication delivery  CCS before trial (no ultrasound data)  EFW <p3 with normal UA PI  Spontaneous birth  Raise in UA PI with >20 centile  No ultrasound information available | 7 (30.4)  7 (30.4)  4 (17.4)  1 (4.3)  3 (13.0)  1 (4.3) |
| CCS = corticosteroids, EFW = estimated fetal weight, UA = umbilical artery, PI = pulsatility index | |

| Table S2. Study outcomes comparing early versus late strategy with maternal indications for delivery excluded. N = 92 patients | | | | |
| --- | --- | --- | --- | --- |
| Outcome | **Early strategy ITT***  **N, % (n=54)** | **Late strategy ITT****  **N, % (n=38)** | **Crude OR**  **(95% CI)** | **Adjusted OR**  **(95% CI)^1^** |
| Primary outcomes | | | | |
| Neonatal mortality (n=18) | 11 (20.4) | 7 (18.4) | 0.88 (0.31 – 2.53) *P*=0.82 | 1.07 (0.33 – 3.45) *P*=0.92 |
| Composite primary outcome (n=46) | 27 (50.0) | 19 (50.0) | 1.00 (0.44 – 2.30) *P*=1.00 | 1.28 (0.45 – 3.65)  *P*=0.65 |
| Secondary outcomes | | | | |
| NEC stage two or more (n=11) | 6 (11.1) | 5 (13.2) | 1.21 (0.34 – 4.30)  *P* =0.77 | 1.30 (0.35 – 4.78)  *P*=0.70 |
| IVH grade three or more (n=2) | 2 (3.8) | 0 (0.0) | 0.0 (0.00 – 0.00)  *P* =1.00 | 0.0 (0.00 – 0.00)  *P*=1.00 |
| Moderate or severe BPD (n=26) | 15 (27.8) | 11 (28.9) | 1.08 (0.42 – 2.81) *P*=0.87 | 1.36 (0.42 – 4.34) *P*=0.61 |
| ROP requiring laser therapy (n=8) | 6 (11.1) | 2 (5.3) | 0.80 (0.12– 5.40) *P*=0.82 | 0.71 (0.06 – 8.68) *P*=0.79 |
| RDS (n=55) | 32 (59.3) | 23 (60.5) | 1.05 (0.45 – 2.46) *P*=0.90 | 1.25 (0.40 – 3.87) *P*=0.71 |
| Sensitivity analyses of neonatal outcomes when comparing early and late strategy based on intention to treat approach where only fetal indication of induction of delivery were analyzed, excluding delivery on maternal indications.  Primary outcome: composite of neonatal mortality, moderate or severe BPD, IVH grade >2, NEC Bell stage >1 and ROP with laser therapy OR= Odds ratio. NEC= necrotizing enterocolitis, IVH= intraventricular hemorrhage, BPD= bronchopulmonary dysplasia, ROP= retinopathy of prematurity, RDS= respiratory distress syndrome.  * Early strategy ITT: corticosteroids when UA pi >p95 or MCA pi <p5, based on intention to treat approach  **Late strategy ITT: corticosteroids when absent/reversed end diastolic flow of umbilical artery, based on intention to treat approach  ^1^Adjusted for gestational age at delivery and birthweight. | | | | |

| Table S3. Study outcomes comparing early versus late strategy, per protocol approach. N = 97 patients | | | | |
| --- | --- | --- | --- | --- |
| Outcome | **Early strategy PP***  **N, % (n=41)** | **Late strategy PP****  **N, % (n=56)** | **Crude OR**  **(95% CI)** | **Adjusted OR**  **(95% CI)^1^** |
| Primary outcomes | | | | |
| Neonatal mortality (n=22) | 8 (19.5) | 14 (25.0) | 1.38 (0.52 – 3.67) p=0.53 | 0.97 (0.33 – 2.86) p=0.96 |
| Composite primary outcome (n=51) | 20 (48.8) | 31 (55.4) | 1.30 (0.58 – 2.92) p=0.52 | 0.78 (0.28 – 2.19) p=0.64 |
| Secondary outcomes | | | | |
| NEC stage 2 or more (n=14) | 6 (14.6) | 8 (14.3) | 0.97 (0.31 – 3.05) p=0.96 | 0.81 (0.25 – 2.67) p=0.73 |
| IVH grade 3 or more (n=3) | 1 (2.4) | 2 (3.6) | 1.41(0.12 – 16.08)p=0.78 | 1.77(0.14 – 22.49) p=0.66 |
| Moderate or severe BPD (n=29) | 13 (31.7) | 16 (28.6) | 0.90 (0.36 – 2.26) p=0.83 | 0.52 (0.16 – 1.64) p=0.26 |
| ROP requiring laser therapy (n=9) | 4 (9.8) | 5 (8.9) | 0.97 (0.19 – 5.03) p=0.97 | 0.22 (0.21 – 2.28) p=0.21 |
| RDS (n=62) | 26 (63.4) | 36 (64.3) | 1.04 (0.45 – 2.40) p=0.93 | 0.66 (0.20 – 2.18) p=0.50 |
| Sensitivity analyses of neonatal outcomes when comparing early and late strategy based on per protocol approach.  Primary outcome: composite of neonatal mortality, moderate or severe BPD, IVH grade >2, NEC Bell stage >1 and ROP with laser therapy  OR= Odds ratio. NEC= necrotizing enterocolitis, IVH= intraventricular hemorrhage, BPD= bronchopulmonary dysplasia, ROP= retinopathy of prematurity, RDS= respiratory distress syndrome.  * Early strategy PP: corticosteroids when UA pi >p95 or MCA pi <p5, based on per protocol approach  **Late strategy PP: corticosteroids when absent/reversed end diastolic flow of umbilical artery, based on per protocol approach  ^1^Adjusted for gestational age at delivery and birthweight. | | | | |

Figure S1. Flow diagram of Dutch STRIDER study

|  |
| --- |
| 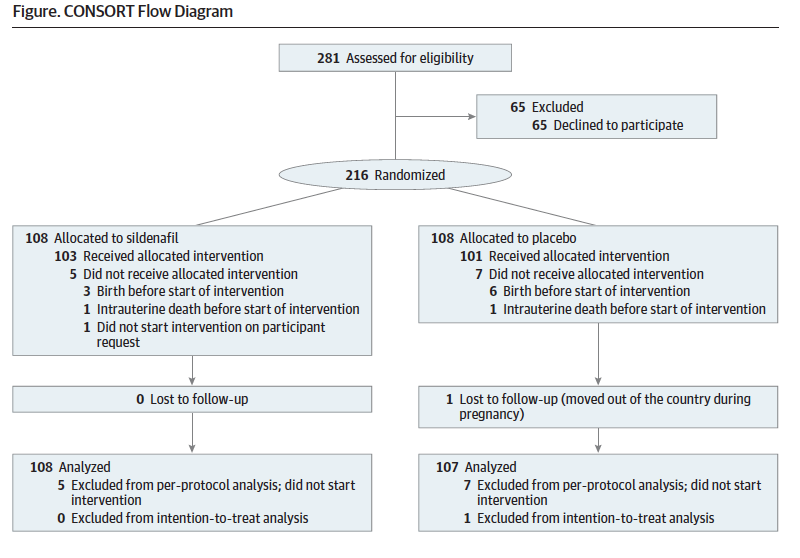 |
| Reprinted with permission from Pels A, Derks J, Elvan-Taspinar A, et al. Maternal Sildenafil vs Placebo in Pregnant Women With Severe Early-Onset Fetal Growth Restriction: A Randomized Clinical Trial. JAMA Netw Open. 2020;3:e205323. |
